# Phosphate deficiency induced biofilm formation of *Burkholderia* on insoluble phosphate granules plays a pivotal role for maximum release of soluble phosphate

**DOI:** 10.1038/s41598-019-41726-9

**Published:** 2019-04-02

**Authors:** Ranjan Ghosh, Soma Barman, Narayan Chandra Mandal

**Affiliations:** 1 Guest Teacher, Mycology and Plant Pathology Laboratory, Department of Botany, Visva-Bharati, Santiniketan, 731235 West Bengal India; 2 National Post Doctoral Fellow, Soil and Agrobio-Engineering Laboratory, Department of Environmental Science, Tezpur University, Napaam, Tezpur, 784028 Assam India; 3 Professor, Mycology and Plant Pathology Laboratory, Department of Botany, Visva-Bharati, Santiniketan, 731235 West Bengal India

## Abstract

Involvement of biofilm formation process during phosphate (P) solubilization by rhizobacterial strains is not clearly understood. Scanning electron microscopic observations revealed prominent biofilm development on tricalcium phosphate as well as on four different rock phosphate granules by two P solubilizing rhizobacteria viz. *Burkholderia tropica* P4 and *B*. *unamae* P9. Variation in the biofilm developments were also observed depending on the total P content of insoluble P used. Biofilm quantification suggested a strong correlation between the amounts of available P and degrees of biofilm formation. Lower concentrations of soluble P directed both the organisms towards compact biofilm development with maximum substratum coverage. Variation in the production of extracellular polymeric substances (EPS) in the similar pattern also suggested its close relationship with biofilm formation by the isolates. Presence of BraI/R quorum sensing (QS) system in both the organisms were detected by PCR amplification and sequencing of two QS associated genes viz. *braR* and *rsaL*, which are probably responsible for biofilm formation during P solubilization process. Overall observations help to hypothesize for the first time that, biofilm on insoluble P granules creates a close environment for better functioning of organic acids secreted by *Burkholderia* strains for maximum P solubilization during P deficient conditions.

## Introduction

Biofilm formation can be defined as a process whereby microbial cells irreversibly attach to and grow on a surface with the production of extracellular organic polymeric matrix resulting in an alteration in the phenotype of the microorganisms with respect to their growth rate and gene transcription. For bacterial organisms, the process of biofilm formation is advantageous as it offers protection to the producing organisms from antibiotics^1^, disinfectants or dynamic environmental conditions. It also helps them to survive in nutrient deficient or oligotrophic conditions^2^. Microbial surface attachment is considered as an important factor during competition for carbon sources and trace elements in the growth limiting conditions especially in the marine environment^3^. About 99% of the world population of bacteria produce diverse biofilm structures during various stages of their growth^4^. Bacterial biofilms may form on a wide variety of surfaces, including living tissues, indwelling medical devices, industrial or potable water system piping, or natural aquatic systems^5^. Different types of noncellular materials like mineral crystals, corrosion particles, clay or silt particles, or blood components, may also be found in the biofilm matrix depending on the environment of biofilm development^5^. Biofilms in natural environments are commonly found on coating around various particles that occur in soils, lake, river and others. In soil, microbial biofilms play a crucial role in the formation and stability of soil aggregates, weathering of different minerals as well as degradation and sequestration of organic carbon^6^. Biofilm formation in drinking water pipe network is a major cause for deterioration of water quality and also for operational problems. It is responsible for the increase of pathogenic bacterial load, decrease of dissolved oxygen, change of taste and odor, and red or black coloration of supplied drinking water^7^. On the other hand, biological wastewater treatment is getting much attention in the recent years and bacterial biofilms impart significant roles for such treatments. Based on bacterial biofilm different types of biofilters have been designed which are useful for successful removal of organic and inorganic pollutants from the wastewater. Another important aspect of microbial biofilms in natural environment is their ability to stabilize sediment which is known as biostabilization. It gives protection to the sediments from erosion. Even after erosion microbial biofilm promote sediment aggregation by altering the floc properties of sediments^8^.

Production of extracellular polymeric substances (EPS) is one of the important features of most of the biofilm forming bacteria and it is considered as the major factor that influences biofilm formation^9^. More than 90% of the dry mass in a mature biofilm is represented by EPS components which include different polysaccharides, proteins, nucleic acids, lipids and other biomolecules^10^. EPS is responsible for bacterial cells adhesion to surfaces and is also responsible for maintaining the three-dimensional architecture and morphology of the biofilm matrix^11,12^. The extensive production of EPS occurs during the specific adhesion stage of biofilm development for successful attachment. EPS also protect the bacterial cells from various stresses like antimicrobials, oxidation and metallic cations. In addition, EPS also helps to retain quorum sensing (QS) signaling molecules, different extracellular enzymes and other metabolic products which ultimately supports cell-cell communication and degradation of substances^13^.

Competition for nutrients and other growth requirements is definitely an important driving force for the biofilm development. Increased cell density favors chemical signals for communicating with the responding cells for social interactions in biofilms. Furthermore, the expression of different adhesins, their cognate receptors, and exopolymeric components by individual cell types within a biofilm community can contribute to overall biofilm development^14,15^. Bacteria communicate with neighbors and monitor their population density by producing and sensing signaling molecules in a process called quorum sensing (QS)^16^. Many bacteria are capable of using a QS mechanism to regulate biofilm formation and other social activities^17^. The concentration of the signaling molecule increases alongside the bacterial population density and, when it reaches a significant level, bacteria respond and modulate target gene expression. In Gram negative bacteria, QS system involves the production and response to a signaling molecule known as acylated homoserine lactone (AHL). On the other hand peptide-based signal molecules like linear and crystallized oligopeptides have been reported in case of Gram-positive bacteria^18^. In addition, some other signaling molecules like gamma-butyrolactones (GBLs) of *Streptomyces* spp. and the A-signaling amino acids of *Myxococcus xanthus* had also been reported and were well studied^19^. The signal molecules are either recognized by specific extracytoplasmic sensor proteins or they can transit across the cell membrane and interact and directly modulates the function of a cytoplasmic target protein. In addition to biofilm formation, QS regulation also plays vital roles in many other processes which include interaction of bacteria with higher organisms, regulation of virulence, conjugation, expression and regulation of genes related to the production of toxins, enzymes, antibiotics and other secondary metabolites^18^. In Gram negative bacteria although QS circuit has been identified for several species but in most of the cases it resembles the canonical QS circuits (*lux* operon) of *Vibrio fischeri*. QS circuit in Gram negative bacteria contains genes homologous to *V*. *fischeri luxI* and *luxR* genes. The product of *luxI* is responsible for biosynthesis of a specific AHL molecule which binds with the receptor protein LuxR. LuxR-AHL complex then activates the target gene transcription^18^.

In the genus *Burkholderia*, QS system has been extensively studied. The species of BCC complex share a conserved QS system known as CepI/R^20^ that produces and responds to *N*-octanoyl homoserine lactone (C8-AHL). CepI/R QS system regulates virulence and several important phenotypes like biofilm formation and siderophore production^21–23^. On the other hand Suárez-Moreno *et al*.^24^ reported BraI/R quorum sensing system which produces and responds mainly to C12-3-oxo-AHL and stringently regulated by a repressor RsaL. Later on, they have also reported that BraI/R QS system is related to EPS production and biofilm formation by some plant associated *Burkholderia* spp.^16^.

Although a large number of soil bacteria were found to solubilize insoluble P but the role of plant associated *Burkholderia* spp. as potent P solubilizers have been reported in the recent years. In the present study two potent P solubilizing bacterial (PSB) strains viz. *Burkholderia tropica* P4 and *Burkholderia unamae* P9 were selected which were isolated from the rhizospheric soil of *Lycopodiella cernua* (L.) Pic. Serm. grown in the P deficient lateritic soil of West Bengal, India^25^. They were found effective to release appreciable amounts of soluble P from insoluble tricalcium phosphate (TCP) or different rock phosphates^25^. Earlier study also suggested a decrease of medium pH by both the isolates during P solubilization process. Although secretion of organic acids by Gram negative bacteria were considered as one of the key mechanisms for inorganic P solubilization, involvement of biofilm formation, EPS production and quorum sensing process during P solubilization were not studied earlier especially in these bacterial species. Thus for finding out the detail mechanisms of P solubilization by the isolates, the present study was focused on their biofilm forming properties during solubilization of insoluble P granules. In addition, EPS production by these organisms was also quantified for understanding their role in biofilm formation as well as in P solubilization processes. Lastly, the presence of QS systems in both the bacterial strains was detected which probably helps in biofilm formation during solubilization of insoluble P by them.

A large number of P solubilizing bacteria has been reported earlier which were able to produce good amount of soluble P *in vitro* but most of the cases their efficiencies in the actual field condition were not satisfactory. Investigation on their biofilm forming properties will be helpful for their proper application as well as for the preparation of suitable formulations to get satisfactory results. In addition, bioprocessing of rock phosphate ores using biofilm forming microorganisms is mentioned earlier^26^ but this field is not well studied till date due to lack of sufficient information. Application of biofilm forming *Burkholderia* spp. for such bioprocessing using appropriate bioreactors will also be a promising approach for the production of soluble P by avoiding conventional acid digestion methods.

## Results

### Biofilm formation on insoluble P granules

**Figure 1:**
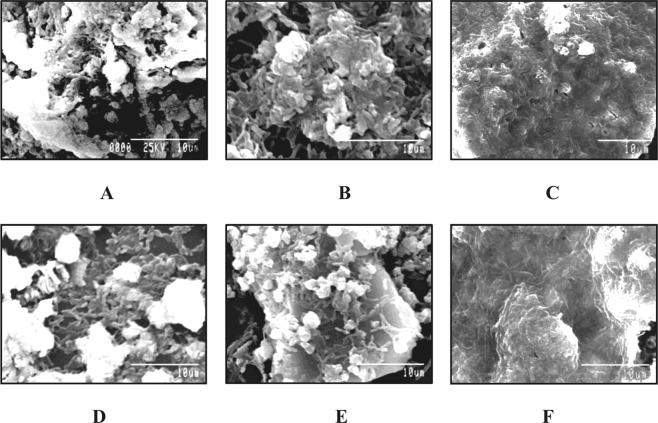
Scanning electron micrographs of biofilm formation by bacterial strains on insoluble phosphate granules. (**A**–**C**) *B*. *tropica* P4; (**D**–**E**) *B*. *unamae* P9; (**A**,**D**) on TCP; (**B**,**E**) on JRP; (**C,F**) on MRP.

In order to observe the biofilm formation by the strains *B*. *tropica* P4 and *B*. *unamae* P9 on the granules of TCP as well as four different rock phosphates viz. Jordan Rock Phosphate (JRP), Purulia Rock Phosphate (PRP), Udaypur Rock Phosphate (URP) and Mussoorie Rock Phosphate (MRP), bacteria were allowed to grow for 48 hours at 28 °C and observed for their physical interactions. Scanning electron microscopic (SEM) observation indicated improved adhesions of bacterial cells on insoluble P granules. Both the strains were effectively produced compact biofilm structures on TCP (Fig. 1A,D) as well as on rock phosphate granules. In addition to firm attachments with insoluble P granules, prominent cell to cell adhesions were also observed for both the strains. Production of extracellular polymeric matrix surrounding those biofilm structures were also observed during SEM study. Although formation of biofilms was found for all the different insoluble P granules, but the extent of biofilm formation varied depending upon the nature of insoluble P granules used in the National Botanical Research Institute Phosphate Growth Medium devoid of Yeast Extract (NBRIY) broth. Among the four different rock phosphates, maximum bacterial attachments as well as cell to cell adhesions were observed on MRP granules for both the isolates (Fig. 1C,F). Very dense extracellular polymeric matrix surrounding the biofilm structures was also found under the higher magnification of SEM for MRP granules in comparison to other rock phosphate used (Fig. 1C,F). On the other hand, comparatively reduced bacterial adherences with thin EPS matrices were noticed for TCP (Fig. 1A,D) and Jordan Rock Phosphate (JRP) granules (Fig. 1B–E).

### Variation in biofilm formation at different concentrations of available phosphate

**Figure 2:**
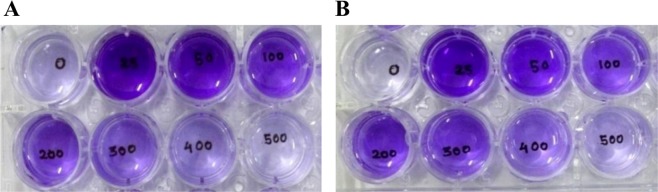
Crystal violet staining of biofilm developed by bacterial strains in the presence of different concentration of K_2_HPO_4_ (0–500 µg/ml). (**A**) *B*. *tropica* P4; (**B**) *B*. *unamae* P9.

**Figure 3:**
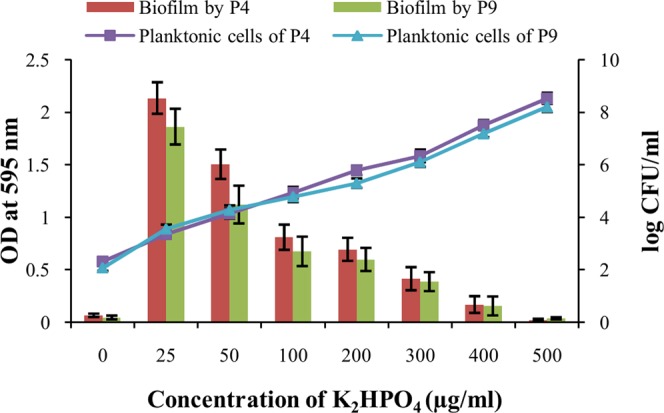
Quantification of biofilm development and planktonic cell growth of bacterial strains at different concentrations of K_2_HPO_4_ (0–500 µg/ml).

Degrees of biofilm formation by the two species of *Burkholderia* were quantified using different concentrations of soluble P (K_2_HPO_4_) in NBRIY medium for finding out the relationship of biofilm formation with available P. When the crystal violet stain was extracted using 33% acetic acid from different wells of 24 wells culture plates, visual differences in biofilm formation at different concentrations of K_2_HPO_4_ were noticed for both the strains (Fig. 2A,B). UV-VIS spectrophotometric measurements of optical densities (OD) of crystal violet solutions indicated maximum biofilm formation by both the strains in the presence of 25 μg/ml of K_2_HPO_4_ (Fig. 3). Maximum OD of 2.135 ± 0.15 and 1.86 ± 0.17 were recorded for *B*. *tropica* P4 and *B*. *unamae* P9 respectively when 25 μg/ml of K_2_HPO_4_ was used. Biofilm formation significantly (P < 0.001) decreased along with increased concentrations of K_2_HPO_4_ for the strains (Fig. 3) and OD of crystal violet solutions suggested almost no biofilm formation by them in the presence of K_2_HPO_4_ at a concentration of 500 μg/ml. In the complete absence of K_2_HPO_4_ in the growth medium, biofilm formations were also negligible for both the organisms. Similar pattern of biofilm formation was also observed for both the strains when the K_2_HPO_4_ was replaced with different concentrations of Na_2_HPO_4_.

### Planktonic cell densities at different concentrations of available P

Planktonic cell densities were also determined at different concentrations of available P in NBRIY medium for both the strains. Counting of colony forming units (CFUs) after plating showed that unlike biofilm formation planktonic cell densities increased significantly (P < 0.001) along with increased concentration of soluble P (K_2_HPO_4_) for both *B*. *tropica* P4 and *B*. *unamae* P9 (Fig. 3). Maximum numbers of planktonic cells were observed when 500 µg/ml of K_2_HPO_4_ was used in NBRIY broth. Reduced planktonic cell densities were also observed in the complete absence of K_2_HPO_4_ in the medium.

### Biofilm morphologies at different concentrations of available P

**Figure 4:**
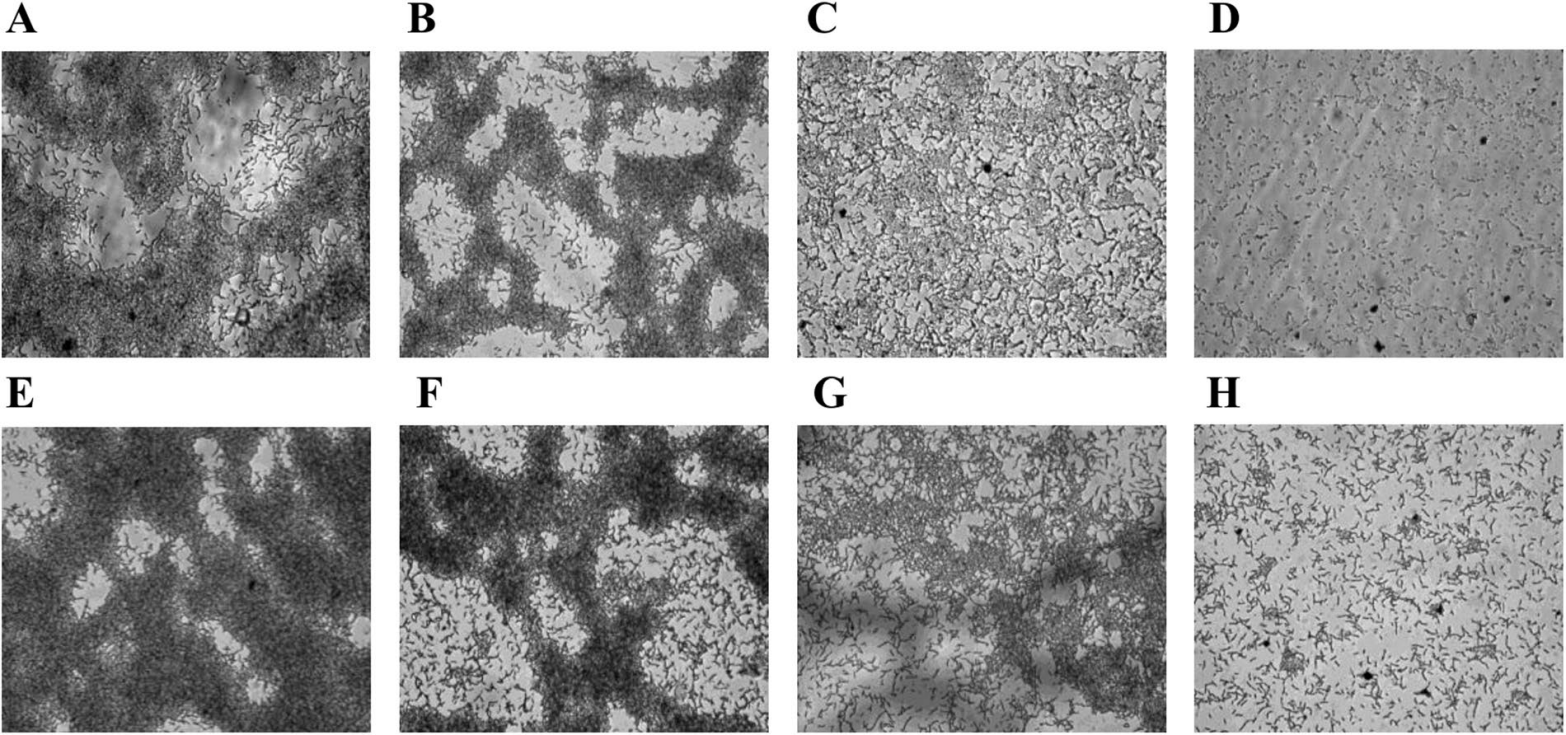
Biofilm morphologies of *B*. *tropica* P4 (**A**–**D**) and *B*. *unamae* P9 (**E**–**H**) at different concentrations of K_2_HPO_4_ (µg/ml) as observed under light microscope: (**A,E**) 25 µg/ml; (**B,F**)- 50 µg/ml; (**C,G**) 100 µg/ml; (**D,H**) 500 µg/ml.

When the polyester films placed in the wells of 24 wells plates were subjected to light microscopic studies after crystal violet staining, prominent structural variations of biofilm morphologies were observed depending on the concentrations of K_2_HPO_4_ used in NBRIY broth. Under light microscope, compact biofilm structures were observed for both the strains at lower concentrations of K_2_HPO_4_ in comparison to higher concentrations. Thick biofilm structures with maximum substratum coverage were observed in the presence of 25 µg/ml of K_2_HPO_4_ (Fig. 4A,E). Both the thickness as well as substratum coverage markedly decreased along with increased concentrations of K_2_HPO_4_ for *B*. *tropica* P4 (Fig. 4B–D) as well as for *B*. *unamae* P9 (Fig. 4F–H). Almost no patchy microcolonies were observed for both the strains above 300 µg/ml of K_2_HPO_4_ (4D,H). On the other hand biofilm structures were also not noticed in the absence of K_2_HPO_4_ in the medium.

### EPS production by bacterial isolates in the presence of different insoluble P

When the EPS production by the strains were quantified in terms of dry weights as well as total carbohydrate contents in the presence of different types of insoluble P in culture medium, maximum EPS production was observed in the presence of MRP followed by URP (Table 1). *B*. *tropica* P4 and *B*. *unamae* P9 produced 5.01 ± 0.29 g/l and 4.86 ± 0.32 g/l of EPS respectively in the presence of MRP as insoluble P source (Table 1). On the other hand EPS production significantly (P < 0.001) decreased in the presence of TCP for both the strain (Table 1). Reduced EPS production was also observed in the presence of JRP and PRP in comparison to MRP. In terms of total carbohydrate also EPS production was almost two times higher in MRP (0.68 ± 0.04 g/l for P4 and 0.59 ± 0.04 g/l for P9) than TCP (0.32 ± 0.04 g/l for P4 and 0.28 ± 0.03 g/l for P9) for both *B*. *tropica* P4 as well as *B*. *unamae* P9 (Table 1).

**Table 1:** EPS production in terms of dry weight as well as total carbohydrate (TC) by isolated bacterial strains during solubilization of different insoluble P.

Insoluble P used in NBRIY broth	EPS production by isolated bacterial strains after 48 hours of incubation			
	*B*. *tropica* P4		*B*. *unamae* P9	
	Dry wt. (g/l)	TC (g/l)	Dry wt (g/l)	TC (g/l)
TCP	2.75 ± 0.25	0.32 ± 0.04	2.68 ± 0.27	0.28 ± 0.03
JRP	2.82 ± 0.27	0.36 ± 0.03	2.92 ± 0.16	0.40 ± 0.07
PRP	3.22 ± 0.22	0.48 ± 0.06	3.45 ± 0.25	0.45 ± 0.05
URP	4.69 ± 0.17	0.54 ± 0.06	4.34 ± 0.29	0.51 ± 0.06
MRP	5.01 ± 0.29	0.68 ± 0.04	4.86 ± 0.32	0.59 ± 0.04

### EPS production in the presence of different concentrations of K_2_HPO_4_

**Figure 5:**
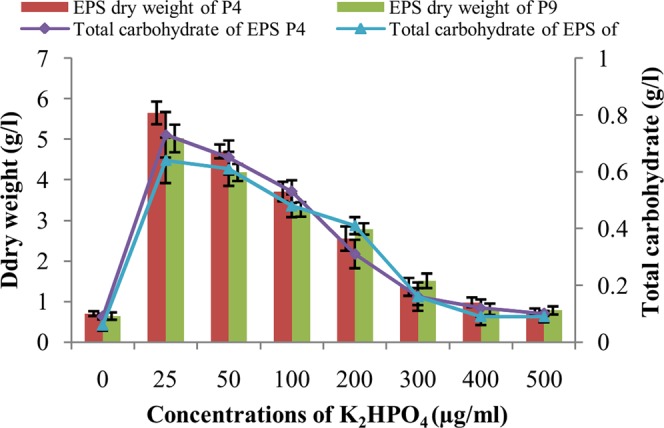
Production of EPS by *B*. *tropica* P4 and *B*. *unamae* P9 in terms of dry weight as well as total carbohydrate content in the presence of different concentrations of K_2_HPO_4_.

Like biofilm formation, EPS production by both the strains also showed similar pattern when culture medium was supplemented with different concentrations of K_2_HPO_4_. Maximum EPS production was observed in the presence of K_2_HPO_4_ at a concentrations of 25 µg/ml for *B*. *tropica* P4 (5.65 ± 0.28 g/l) as well as *B*. *unamae* P9 (5.02 ± 0.34 g/l) which decreased gradually (P < 0.001) along with increased concentrations of K_2_HPO_4_. In terms of total carbohydrate contents also EPS productions by the isolates showed similar patterns (Fig. 5).

### Presence of BraI/R QS system in *B*. *tropica* P4 and *B*. *unamae* P9

**Figure 6:**
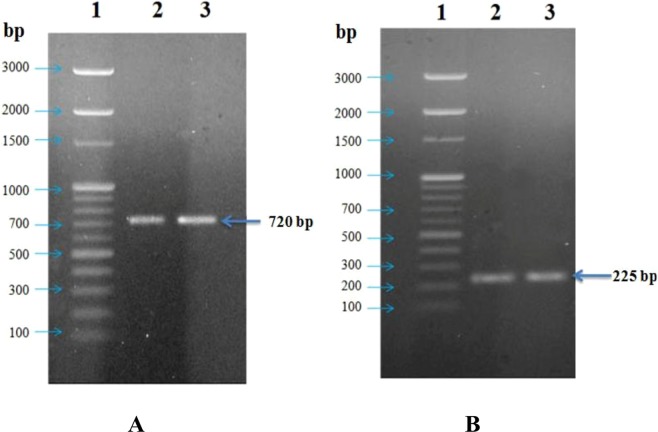
Amplified DNA for *braR* and *rsaL* gene on 1.2% agarose gel: (**A**) lane 1–100 bp DNA ladder; lane 2- *braR* of *B*. *tropica* P4; lane 3- *braR* of *B*. *unamae* P9; (**B**) lane 1–100 bp DNA ladder; lane 2- *rsaL* of *B*. *tropica* P4; lane 3- *rsaL* of *B*. *unamae* P9.

In both the organisms BraI/R QS system was detected by amplifying two genes i.e. *braR* and *rsaL* using the primers described earlier^16^. When the amplified DNA were resolved on 1.2% agarose gel single band of nearly 720 bp for *braR* gene was detected for both *B*. *tropica* P4 as well as *B*. *unamae* P9 (Fig. 6A). On the other hand nearly 225 bp bands were observed for *rsaL* gene of the strains (Fig. 6B). Nucleotide sequence of each gene was generated using forward and reverse sequence data and confirmed as *braR* and *rsaL* gene by BLAST analysis on NCBI GenBank database.

Clustal Omega (1.2.1) multiple sequence alignment program revealed that *braR* of *B*. *tropica* P4 showed 79.50% identity with *B*. *kururiensis* M130 and 93.79% identity with *B*. *unamae* MTI-641^T^ where as *braR* of *B*. *unamae* P9 showed 80.90% identity with *B*. *kururiensis* M130 and 96.12% identity with *B*. *unamae* MTI-641^T^. On the other hand *rsaL* nucleotide sequence of *B*. *tropica* P4 showed 74.32% identity with *B*. *kururiensis* M130 and 85.79% identity with *B*. *unamae* MTI-641^T^ where as the *rsaL* nucleotide sequence of *B*. *unamae* P9 showed 73.63% identity with *B*. *kururiensis* M130 and 84.62% identity with *B*. *unamae* MTI-641^T^.

## Discussion

In environment several microorganisms aggregate at solid-liquid interfaces to form adherent multicellular structures which is generally known as biofilm. During the development of biofilm, bacterial cells attach to each other and often these cells adhere to a solid surface. Sometimes these adherent cells remained embedded within an extracellular matrix generally made up of polysaccharides and other macromolecules. Microorganisms produce biofilm to conquer different environmental stresses such as nutrient limitation, desiccation and predatory grazing^2^. In pathogenic microorganisms, biofilm generally serve as one of the important protecting mechanisms against several antimicrobials^27^. In addition to various drawbacks of microbial biofilm development, there are also several positive aspects^28^. As for example, biofilm formation by several bacterial strains on crop plant rhizosphere confer protection against various fungal diseases^29^.

The present work demonstrated biofilm formation by two plant growth promoting bacterial strains during solubilization of insoluble P. Our initial observations suggested maximum biofilm formation by both the strains after 48 hours of incubation. After 48 hours of incubation, formation of prominent biofilm structures by both the strains of *Burkholderia* on all the insoluble P granules was observed using scanning electron microscopic technique. Both the strains were found to attach firmly to the surfaces of rock phosphate granules which can withstand even after repeated washing. Surface roughness of rock phosphate granules probably plays major role for their stable attachments. Cell surface hydrophobicity, presence of different appendages and degrees of EPS production have also been considered as important factors for bacterial attachments^30^. It has been reported that three different types of forces viz. hydrogen bonds, electrostatic interaction and london dispersion forces are involved in biofilm formation process^31^. In case of *B*. *mallei* and *B*. *pseudomallei*, role of trimeric autotransporter adhesins has also been suggested for their attachment and biofilm formation during environmental as well as virulence-associated interactions^32,33^.

Although maximum biofilms formation by the isolates was detected after 48 hours of incubation, maximum release of soluble P was detected after 72 hours of incubation for most of the insoluble P used^25^. When TCP was used as sole P source, after 48 hours of incubation at 28 °C, 370.69 ± 8.79 µg/ml and 463.03 ± 15.17 µg/ml of soluble P were released by *B*. *tropica* P4 and *B*. *unamae* P9 respectively. After 72 hours of incubation the amount of soluble P increased to 580.56 ± 13.38 µg/ml and 517.12 ± 17.15 µg/ml for *B*. *tropica* P4 and *B*. *unamae* P9 respectively^25^. The decrease of medium pH by both the strains (pH 3.35 ± 0.20 by P4 and pH 3.50 ± 0.17 by P9 after 72 hours of incubation) was also correlated with release of soluble P in the medium. Initial pH of 6.5–7.0 was found effective for maximum bacterial growth and they decrease medium pH to less than pH 3.5 within 72 hours of incubation. They were also able to survive in these lower pH with sufficient CFUs. The current observation suggested that biofilm formation by the strains within 48 hours of incubation probably creates a close environment to secrete organic acid by them which was responsible for maximum release of soluble P after 72 hours of incubation. Most of the PSB solubilize inorganic P by secreting different types of organic acids like gluconic acid, 2-keto gluconic acid, citric acid, malic acid, oxalic acid, lactic acid and others^34,35^. IR spectrophotometric analysis of partially purified solubilizing principles suggested the production of carboxylic acids by both the isolates. Appearances of peaks at 1620.09 cm^−1^ and 3382.91 cm^−1^ for P4 (see Supplementary Fig. S1) and at 1757.03 cm^−1^ and 3492.85 cm^−1^ for P9 (see Supplementary Fig. S2) suggested the presence of C=H stretch and O-H stretch respectively for carboxylic acids. The major soluble form of mineral P is the monovalent anion phosphate H_2_PO_4_^−^ which generally produces at lower pH. Organic acid produced by PSB decreases the pH of surrounding environment which ultimately helps to release P ions by H^+^ substitution for the cation bound to P^36^. Gram negative bacteria generally produce gluconic acid and 2-keto gluconic acid during P solubilization process. Gluconic acid is produced mainly in the bacterial periplasmic space by direct oxidation of glucose with the help of pyrrolo quinoline quinone (PQQ) dependent membrane bound enzyme glucose dehydrogenase (m-GDH). PQQ act as a redox cofactor for the enzyme m-GDH^37^. Gluconic acid may further oxidized to 2-keto gluconic acid by the enzyme gluconate dehydrogenase^38^.

Comparison between the degrees of biofilm formation on different insoluble P granules revealed maximum biofilm formation by both the organisms on MRP granules. It has been reported earlier that P content in MRP was low (17% P_2_O_5_) in comparison to other rock phosphates^39^. Therefore, release of appreciable amount of soluble P from low P content MRP granules required maximum efforts, and that may be fulfilled by producing maximum biofilm coverage. On the other hand JRP contained 34% P_2_O_5_, and the strains *B*. *tropica* P4 and *B*. *unamae* P9 produced 514.80 ± 11.33 µg/ml and 464.11 ± 12.15 µg/ml of soluble P by using JRP^25^. Therefore, less biofilm development may also be correlated with its high P content and easy release of soluble P in appreciable amount.

By assuming the role of available P on the extent of biofilm formation by the isolates, quantification of biofilm formation was performed in the presence of different concentrations of soluble P (K_2_HPO_4_). Maximum biofilm formation by both the strains under P limitation conditions, suggested that the P solubilizing bacterial strains attach to the surfaces of insoluble P granules and release more soluble P for overcoming the stress generated by P limitation. Upregulation of alkaline phophatase activity in phosphorous limited biofilm has been reported by Huang *et al*.^40^. P limitation also enhanced the biofilm formation of plant pathogenic bacteria *Agrobacterium tumefaciens*^2^. Reduced planktonic cell densities at lower K_2_HPO_4_ concentrations also supported the positive correlation between P limitation and development of bacterial biofilm. On the other hand in case of *Escherichia coli* impairment in biofilm formation was observed in the presence of high concentration of (>25 mM) of P in the culture medium. Addition of higher concentration of P to the medium caused disassembly of 24 hours preformed biofilm^41^. It has been found that PolyP degradation is an important factor for biofilm formation at stationary phase. Excess P concentration in the culture media was responsible for high PolyP level up to stationary phase which ultimately impaired biofilm formation in *E*. *coli* in a PhoB dependent manner^42^. Effect of environmental P concentration has also been noticed in the biofilm formation pattern on uropathogenic *E*. *coli* isolates and implication of P as a possible physiological signal was highlighted to regulate biofilm phenotype in *E*. *coli* species^43^. It has also been reported that bacterial stringent response plays important roles in response to low level of specific nutrients including amino acids, glucose, phosphate, iron and fatty acids^44^. It also imparts significant function in antibiotic tolerance and biofilm formation. Some intercellular alarmons viz. guanosine tetraphosphate and guanosine pentaphosphate [collectively known as (p)ppGpp] are considered as universal signal molecules during that type of response^45^. Significant increase in (p)ppGpp levels was described in several stress conditions and depended on the presence of two ribosome associated protein RelA and SpoT that act as (p)ppGpp synthase and hydrolases. (p)ppGpp tunes the RNA polymerase for specific regulation of promoters of the gene associated with bacterial stress tolerance^46^. Use of other growth requirements in same concentrations during quantification experiments strongly suggested involvement of P source in biofilm formation processes for both the strains of *Burkholderia*. On the other hand, similar patterns of biofilm formation after alteration of phosphate source (K_2_HPO_4_) with Na_2_HPO_4_ clearly demonstrated that biofilm formation process was dependent only on the concentration of P and independent on the nature of P source. This type of independency during biofilm formation on the nature of P source was also observed for plant pathogenic bacteria *Agrobacterium tumefaciens*^2^.

Although maximum biofilm formation by the strains P4 and P9 was observed in the presence of 25 µg/ml of K_2_HPO_4_, both of them were unable to form biofilm in the absence of soluble P due to reduced bacterial growth in the absence of essential macroelement phosphorous. Morphological variations of biofilm structures under light microscope also supported their tendencies towards biofilm development at P limitation conditions. Little bacterial growth was detected in the absence of K_2_HPO_4_ due to trace amount of soluble P contamination in other nutrients supplied. There are four major steps in biofilm formation process which include attachment to the surface, growth of bacteria, biofilm maturation and dispersion of cells from matured biofilm. Among them, initial attachment and bacterial growth impart critical role for overall biofilm development and can be considered as rate limiting steps in biofilm formation process. To support the sufficient bacterial growth and biofilm formation, 25 µg/ml of K_2_HPO_4_ was supplied in the NBRIY medium. When the experiments were performed using rock phosphate granules, rock phosphates were used after repeated washing to remove trace amount of soluble P. The organisms were able to form biofilms after initial solubilization of rock phosphates to fulfill their P requirement. Maximum biofilms development after 48 hours also supported their initial requirement of P for maximum growth and biofilm development which ultimately help for maximum (>500 µg/ml) release of soluble P. For better yield, crop plants require more than 60 ppm of soluble P but in agricultural soil P concentration is very low (nearly 1 ppm) which cannot fulfill the need of crop plants^47^. The present observation suggested that the *Burkholderia* strains can be used in combination with rock phosphates to fulfill the P demand in agricultural soil for better crop production.

The biosynthesis of EPS serves many functions like promotion of bacterial attachments to solid surfaces and formation as well as maintenance of microcolony and mature biofilm structure^6^. In addition, EPS enhances the resistance of biofilm structures to environmental stresses and sometime also help the bacteria for accumulating the nutrients^48^. In the present endeavor, increase of EPS production along with biofilm development by both the strains, strongly indicated the direct involvement of EPS during biofilm formation at P deficient conditions. Involvement of EPS production in biofilm formation has been reported by several workers for various bacterial species^9^. Quantification in terms of dry weight and total carbohydrate content also suggested variation in EPS production in the presence of different insoluble P as observed during SEM study. Maximum amount of EPS production in the presence of MRP, directs the bacterial organisms to develop more condensed biofilm structures to release sufficient soluble P from MRP granules. Due to sufficient P content in TCP and JRP, bacterial organisms faced less difficulties to produce significant amount of soluble P, which ultimately suppressed the necessities of EPS production for the development of condensed biofilm structures. Highest degrees of EPS production in the presence of lowest concentration of K_2_HPO_4_ also suggested its strong correlation with available P. According to Chunha *et al*.^49^, EPS production was not essential in *Burkholderia cepacia* complex for biofilm initiation but it plays major role for the establishment of thick biofilm structures. Significant amount of EPS production was also reported for four bacterial strains under the genus *Enterobacter*, *Arthrobacter* and *Azotobacter* having TCP solubilization potential^50^. A branched acetylated heptasaccharide known as cepacian was detected in different members of *Burkholderia* as the major exopolysaccharide which is important for their survival at different environmental conditions like desiccation, metal ion stress etc.^51^.

In most of the Gram negative bacteria AHL dependent quorum sensing system plays significant role in biofilm formation. In the present study presence of BraI/R QS system was detected in *B*. *tropica* P4 and *B*. *unamae* P9. As the primer sets were effective to amplify the *braR* and *rsaL* gene from 20 species of *Burkholderia*^24^, therefore they were used to detect the presence of BraI/R QS system in both the bacterial strains. Suárez-Moreno *et al*.^16^ also reported that biofilm formation and EPS productions were regulated by BraI/R QS system in some plant associated *Burkholderia* spp. Transcriptome analysis also suggested that BraI/R regulon is species specific and associated with EPS production in *Burkholderia xenovorans* LB400 and *B*. *phymatum* STM81^52^. Involvement of similar type of QS system was also reported in regulation of siderophore production in *B*. *cepacia*^21,22^. Although no such direct correlation between siderophore production and P solubilization have been reported, better iron phosphate solubilizing potential was observed in siderophore producing *B*. *cepacia*^25^. In the present observation it was found that biofilm formation and EPS production by *B*. *tropica* P4 as well as *B*. *unamae* P9 were highly correlated with available P in the medium. Although further studies are required, the present observation suggested that there may be some positive relation of this QS system which regulates the biofilm formation or EPS production during P solubiliziation by the isolates.

In developing countries like India, China, Bangladesh, Vietnam, Cambodia, Laos and others, agriculture sector is considered as one of the important pillars of economy for their development. In terms of agricultural aspects phosphorus is the second major macronutrient that required for growth and development of crop plants. The high cost of chemical fertilizers is a great problem for developing countries^53^. Environmental pollution is another major concern regarding the excessive application of chemical fertilizers. Application of P solubilizing microbes is considered as a suitable alternative in this regard. The observed correlation between biofilm formation and P solubilizing process will be helpful to apply the species of *Burkholderia* along with rock phosphate granules in the appropriate field condition as biofertilizer to fulfill the P demands of crop plants for their better production. Suitable formulation can be prepared based on present study for their proper application. When the P solubilizing potential of the strains were studied at different temperatures, both the strains were able to release sufficient amount of soluble P from TCP at low temperature like 20 °C (>330 µg/ml) as well as high temperature like 40 °C (>300 µg/ml)^25^ which also confirmed their applicability at temperature fluctuating countries. Both the organisms were also found to grow in comparatively higher pH (pH 9) and were able to produce prominent zones of P solubilization which also indicated their applicability in highly basic soils. On the other hand, bioprocessing of rock phosphate for the production of soluble P may provide an energy efficient and environmentally favorable alternative in comparison to current acid digestion technology for industrial production of P fertilizers^26^. Selective release of soluble P by bacterial biofilms from rock phosphates also reduces the extraction of undesirable ore contaminants like radioactive toxic materials. Biofilm forming properties of the isolates will be applicable to design proper bioreactors for the processing of rock phosphates following bioprocessing technology. Fixed bed biofilm reactors and contact based biofilm reactors can be used with suitable modification for their application in this purpose. Based on our study it can be suggested that rock phosphates will be used as solid phase and lower concentration of soluble P will be maintained within the bioreactors for the development of maximum biofilm and release of soluble P. In addition to bioprocessing of rock phosphates, the isolates can also be used for P recycling from the sludge produced during wastewater treatment. During chemical processing of wastewater a huge amount of sludge with precipitated P is produced. This sludge with high P contents is considered as a cheap source of P especially in developing countries with limited rock phosphate sources. Effective P solubilizing microorganisms can be used to solubilize and recycle the P from the wastewater sludge which will fulfill the P demands in the agricultural fields of developing countries. Therefore the isolates *B*. *tropica* P4 and *B*. *unamae* P9 may also be helpful for bioprocessing of sludge for releasing soluble P which can be used as biofertilizer for better crop production.

## Methods

### Microbial strains and culture condition

Two potent P solubilizing bacterial strains viz. *Burkholderia tropica* P4 and *B*. *unamae* P9, isolated from the rhizospheric soil of *Lycopodiella cernua* (L.) Pic. Serm^25^ were taken in the present study. The bacterial strains P4 and P9 were previously identified on the basis of 16 S rDNA sequence homologies^25^. Both the strains were maintained in NBRIY medium and preserved at 4 °C. Glycerol stocks were also prepared for long time preservation of the strains. Both the strains were grown at 28 °C in BOD incubator (Instrumentation India, Kolkata, India).

### Study of biofilm formation on insoluble P granules by P4 and P9

Biofilm formation during P solubilization by both the strains viz. *B*. *tropica* P4 and *B*. *unamae* P9 were studied using TCP as well as four different rock phosphates viz. JRP, PRP, URP and MRP.

### Culture conditions and development of bacterial biofilm

Both the bacterial strains were grown in nutrient broth at 28 °C with mild shaking (120 rpm). 100 µl of starter cultures (OD_620nm_ = 0.5) were added to 100 ml of NBRIY broth in 250 ml Erlenmeyer flasks supplemented with different insoluble P at a concentration of 5 g/l. Sterilized polyester chips (2 cm × 2 cm) were placed within each flask and incubated at 28 °C without shaking. At every 24 hours of intervals polyester chips were removed carefully from all the flasks without any agitation and then slowly washed with sterilized distilled water for removing the planktonic cells and finally air dried aseptically in front of laminar air flow (Klenzaids, Mumbai, India).

### Study of bacterial biofilms under scanning electron microscope

All the polyester chips containing layers of insoluble P granules and bacterial cells were prepared for SEM study^54^. They were prefixed with 2% glutaraldehyde in 20 mM Na-P buffer (pH 6.5) plus 5% dimethyl sulphoxide (DMSO) for 30 minutes. After pre-fixation the samples were gently washed with sterilized distilled water and post fixed with osmium tetraoxide dissolved in 50 mM Na-P buffer (pH 6.5). All the samples were then dehydrated using a series of alcohol grades (30–100%) retaining them for 10 minutes in every dilution. The polyester chips were cut into small pieces (4 mm × 4 mm) and placed on metal stubs. They were coated with gold using an ion sputter (Coater IB-2, Gike Engineering, Japan) and finally observed using SEM (HITACHI S-530, Japan). Biofilm formation patterns by the strains on TCP as well as on four different rock phosphates were compared.

### Study of biofilm formation by isolated bacterial strains in relation to available P

In order to ascertain the relations of biofilm formation by P4 and P9 with phosphate availability in their surrounding environment, the insoluble P source of the NBRIY medium was replaced with different concentrations of soluble P i.e. dipotassium phosphate (K_2_HPO_4_). In the presence of different amounts of soluble P, biofilm formation were quantified, biofilm morphologies were studied and the planktonic cell densities were also determined to correlate the biofilm forming tendencies of the isolates in relation to available P.

### Quantification of biofilm formation

Two ml of NBRIY broth (pH 7.0) supplemented with different concentrations of K_2_HPO_4_ were taken in separate wells of 24 wells polystyrene cell culture plates and were inoculated with 20 μl of bacterial culture (OD_620nm_ = 0.5). Uninoculated wells for each concentration were treated as control. All the plates were then kept undisturbed in a BOD incubator (Instrumentation India, Kolkata, India) at 28 °C for 48 hours. After that broth were decanted from each well without disturbing the biofilms and the wells were washed twice with sterilized distilled water carefully to remove the planktonic cells. After air drying in front of laminar airflow, Two ml of 0.1% crystal violet solution was added to each well^55^. After 10 minutes of incubation at room temperature, the stain was removed from the wells and the plates were finally washed with sterilized distilled water for 3–4 times and again air dried. One ml of 33% acetic acid was applied to each well and then incubated for 30 minutes for extracting the crystal violet from adhered cells^56^. Optical densities were then measured at 595 nm using UV-VIS spectrophotometer (SPECTRASCAN UV-2700, Thermo Scientific, India). For confirming the role of P concentration on biofilm formation process, similar quantification experiments were also performed by replacing the P source (K_2_HPO_4_) with Na_2_HPO_4_.

### Estimation of planktonic cell densities

For determining the planktonic cell densities, 100 μl of bacterial culture was taken from each well before decanting the broth and sprayed on nutrient agar (NA) plates after serial dilution using sterilized distilled water. Plates were incubated at 28 °C for 24 hours and numbers of CFUs were counted.

### Study of biofilm morphologies

In order to observe the morphological variation in biofilm structures in relation with available P, sterilized polyester chips (2 cm × 1 cm) were placed in slanting position within the wells of cell culture plates. Two ml of NBRIY broth (pH 7.0) containing different concentrations of K_2_HPO_4_ was added to each well and inoculated with bacterial strains. Plastic chips were removed carefully after 48 hours of incubation at 28 °C, washed slowly with sterilized distilled water and air dried. All the polyester chips with bacterial biofilm were stained with 0.1% crystal violet, air dried and finally observed under light microscope (LEICA DM2500, Germany).

### Study of EPS production by bacterial strains

EPS production by both the strains were studied in 100 ml of NBRIY broth either supplemented with different insoluble P or soluble P i.e. K_2_HPO_4_. EPS production was quantified in terms of dry weight as well as total carbohydrate content. Bacterial cultures were centrifuged at 1000 rpm for 20 minutes to get the cell free supernatants (CFS). EPS from CFS was precipitated by adding same volume of ice cold ethanol followed by incubation at 4 °C for overnight^57^. Precipitated EPSs were separated by centrifugation at 7000 rpm for 20 minutes. To measure the dry weight, EPSs were taken in petriplates and dried at 60 °C. On the other hand for measuring the total carbohydrate, precipitated EPSs were resuspended in 5 ml of sterilized distilled water. One ml of sample was taken to measure the total carbohydrate by phenol sulfuric acid method^58^ using sucrose as standard.

### EPS production in the presence of different insoluble phosphates

EPS production by both the bacterial strains were studied in the presence of TCP as well as four different rock phosphates viz. JRP, PRP, MRP and URP. Each bacterial strain was inoculated separately to 100 ml NBRIY broth (pH 7.0) containing different insoluble P (5 g/l) in 250 ml Erlenmeyer flasks. The EPS produced by the strains were quantified after 48 hours of incubation at 28 °C. EPS production by all the bacterial isolates were quantified in terms of dry weight as well as total carbohydrate contents as mentioned.

### EPS production in the presence of different concentrations of soluble P

In order to determine the relation of EPS production by bacterial strains with available P in the media EPS production were also quantified in the presence of different concentrations of K_2_HPO_4_. Similar to biofilm formation experiments, insoluble P source of NBRIY broth (pH 7.0) was replaced by K_2_HPO_4_. Each bacterial strain was inoculated separately to 100 ml NBRIY broth (pH 7.0) containing different concentrations of K_2_HPO_4_ (0, 25, 50, 100, 200, 300, 400, 500 μg/ml) in 250 ml Erlenmeyer flasks and incubated at 28 °C with mild shaking (120 rpm). Dry weight and total carbohydrate contents of EPS were determined after 48 hours of incubation as mentioned.

### Detection of bacterial quorum sensing system

The presence of BraI/R quorum sensing system, found in many plant associated *Burkholderia* sp.^24^ was detected for the strain P4 and P9 by PCR amplification followed by sequencing of genes (*braR and rsaL*) related with QS systems. *braR* gene of both of the bacterial strains (*B*. *tropica* P4 and *B*. *unamae* P9) was amplified at an annealing temperature of 62 °C. Following primer pairs^24^ were used for the amplification of *braR* gene for both the bacterial strains- pQEbraRfw: 5′-GGGGATCCTCGCCGATACTGGCCGCATC-3′ pQEbraRrv: 5′-GGGAAGCTTTCAGCCCGGATCTATAAGGCC -3′. On the other hand *rsaL* gene of both the organisms was also amplified at an annealing temperature of 48 °C. Following primer pairs^17^ were used for the amplification of this gene for the strains- braLFw: 5′-TTGTTGAAATAAAGTCCCAG-3′ braLRv: 5′-CTGGAAAATCACTGGCA-3′. Amplified DNA was resolved on 1.2% agarose gel and purified using Xcelgen DNA Gel/PCR Purification Miniprep kit following manufacturer instructions. The purified PCR products were sequenced from Xcelric Genomics, India. Sequencing were done in forward and reverse direction in separate reactions. Each reaction mixtures were prepared with template DNA, specific primer, water and BigDye sequencing buffer and BigDye Terminator v3.1 Ready Reaction Mix (Applied Biosystems) of required volumes. Each reaction was heated to 96 °C for one minute, followed by 25 cycle at 96 °C for 10 sec, 50 °C for 5 sec and 60 °C for 4 min. The product was purified following ethanol precipitation method and pelleted by centrifugation. The pellet was washed with 70% ethanol and then air dried. The air dried samples were rehydrated using 15 µl of formamide and then used for sequencing using ABI 3730xl genetic analyzer (Applied Biosystems, USA). Sequence alignment program BioEdit (http://www.mbio.ncsu.edu/BioEdit/bioedit.html) was used for assembling the nucleotide sequences. Nucleotide sequences were used for carrying out BLAST with the NCBI GeneBank database (http://www.ncbi.nlm.nih.gov/) and aligned by Clustal Omega (1.2.1) multiple sequence alignment program^59^.

### Statistical analysis

All the experiments were repeated and the data represented were means of at least three replicates. Means and standard deviations were calculated by using Microsoft Excel program version 2007. Student t-test was performed to test the significance of difference between the mean values of EPS production (both in terms of dry weight and total carbohydrate) in the presence of different insoluble P sources. Single factor ANOVA was performed at 0.05 significant levels to assess the variance of biofilm formation, planktonic cell densities and EPS production in the presence of different concentrations of soluble P.

## Supplementary information

Supplementary Information
