# Supplementary material for: Phosphate deficiency induced biofilm formation of Burkholderia on insoluble phosphate granules plays a pivotal role for maximum release of soluble phosphate
Source: Sci Rep. 2019 Apr 2;9:5477. doi: 10.1038/s41598-019-41726-9 (PMC6445130; doi:10.1038/s41598-019-41726-9)
Supplement: Supplementary file 1 — Supplementary Information [file 41598_2019_41726_MOESM1_ESM.pdf]

Supplementary information (Scientific Reports)

Phosphate deficiency induced biofilm formation of *Burkholderia* on insoluble phosphate granules plays a pivotal role for maximum release of soluble phosphate

Ranjan Ghosh, Soma Barman, Narayan Chandra Mandal

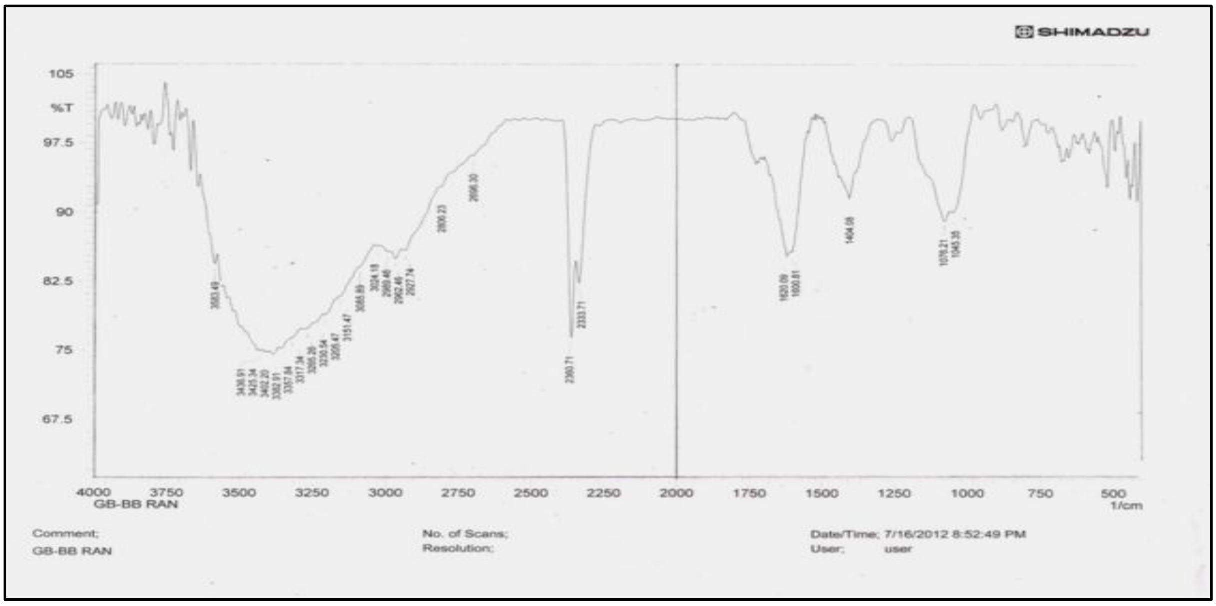

Supplementary Figure S1. IR spectrum of P solubilizing principle of *B. tropica* P4 derived after DOWEX 50 H^+ column chromatography.

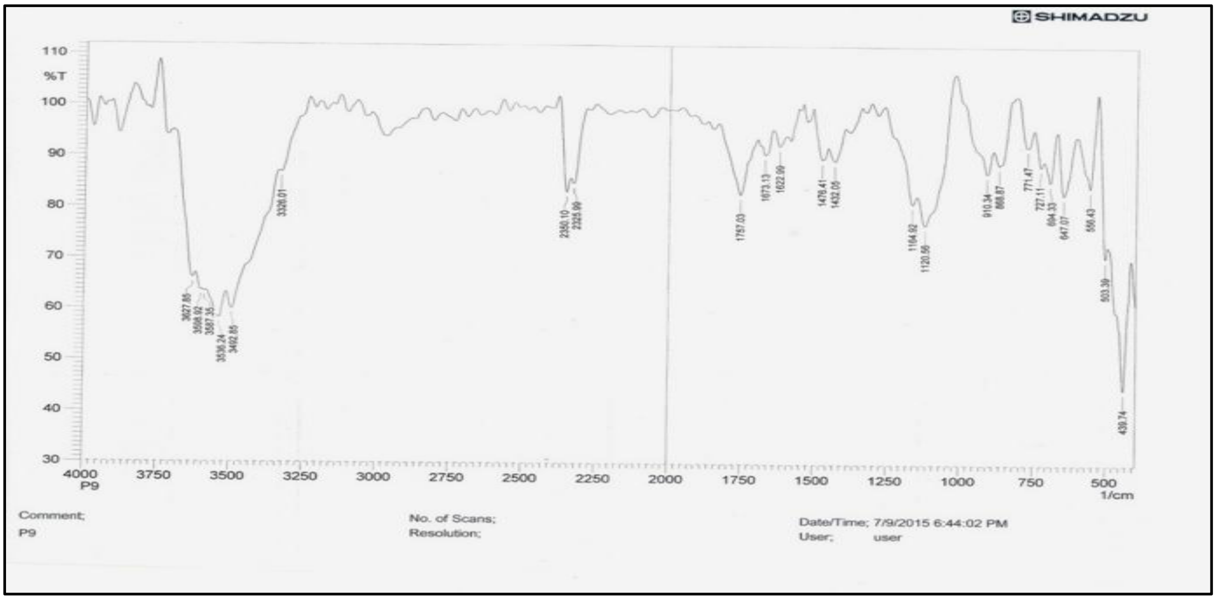

Supplementary Figure S2. IR spectrum of P solubilizing principle of *B. unamae* P9 derived after DOWEX 50 H^+ column chromatography.
